# Global Burden of Alopecia Areata and Associated Diseases: A Trend Analysis From 1990 to 2021

**DOI:** 10.1111/jocd.70076

**Published:** 2025-02-27

**Authors:** Jia Zhou, Luling Liang, Hanlin Zhang, Mingjuan Liu, Zhou Zhu, Ling Leng, Jun Li

**Affiliations:** ^1^ Department of Dermatology, State Key Laboratory of Complex Severe and Rare Diseases Peking Union Medical College Hospital, Chinese Academy of Medical Sciences and Peking Union Medical College, National Clinical Research Center for Dermatologic and Immunologic Diseases Beijing China; ^2^ Stem Cell and Regenerative Medicine Lab, State Key Laboratory of Complex Severe and Rare Diseases, Institute of Clinical Medicine, Translational Medicine Center Peking Union Medical College Hospital Beijing China

**Keywords:** alopecia areata, comorbidity, global burden of disease, hair loss, incidence, psychological impact

## Abstract

**Background:**

Alopecia areata (AA) is a chronic autoimmune disorder characterized by non‐scarring hair loss, affecting approximately 2% of the global population. Its etiology involves genetic, immunological, and environmental factors, with significant psychological impacts such as anxiety and depression. However, international trends and comorbidity associations remain poorly understood.

**Aims:**

This study aims to analyze global trends in the incidence and prevalence of AA and assess its associations with major comorbidities across different demographic and regional groups.

**Patients/Methods:**

Using data from the Global Burden of Disease Study (GBD) 2021, AA incidence, prevalence, and years lived with disability (YLDs) from 1990 to 2021 were analyzed by age, sex, geographic region, and socioeconomic indices (SDI and HDI). Temporal trends were evaluated with Pearson's correlation and Joinpoint regression, and comorbidities were assessed against 22 conditions.

**Results:**

From 1990 to 2021, global AA incidence increased in absolute terms, though age‐standardized incidence rates (ASIR) slightly declined. Incidence was highest in North America, Southeast Asia, and Australia, with females and individuals aged 30–34 most affected. Significant associations were found with atopic dermatitis, iron deficiency, and depressive disorders, with regional variations in comorbidity patterns.

**Conclusions:**

This study highlights rising AA cases with stable ASIRs, reflecting improved awareness and reporting. Findings underscore the complex interplay of socioeconomic factors, healthcare access, and AA burden. Further research should investigate mechanisms underlying comorbidities and guide targeted interventions to mitigate the physical and psychological impacts of AA.

## 1 Introduction

Alopecia areata (AA) is a chronic and non‐scarring form of hair loss that can unpredictably affect any hair‐bearing area. Commonly, the presentation is patchy hair loss on the scalp. Epidemiological studies suggest that AA affects approximately 2% of the population globally in a lifetime, more commonly diagnosed in women over 45 years [1, 2]. The safety and efficacy of current therapeutic approaches for alopecia require further experimental validation [3]. An analysis of the US Census population showed the highest prevalence of AA among adults aged 30–49 and Asian individuals, with notably lower rates observed in White patients [4].

AA has a profound impact on psychological health and overall quality of life. Studies have shown that individuals with AA frequently experience anxiety, depression, and lowered self‐esteem, with some reporting a sense of loss of control over their bodies. The social stigma associated with visible hair loss can lead to social isolation and reduced participation in daily activities [5]. AA represents a dermatological concern and a systemic and psychological burden for affected individuals.

The etiology of AA is complex and warrants further investigation. Genetic predispositions play a notable role, with increased risk observed among family members and individuals with certain genetic markers, such as specific IL‐1 receptor polymorphisms [6]. AA is also widely considered an autoimmune condition in which T lymphocytes target hair follicles, compromising the immune‐privileged environment and leading to hair loss [7]. Psychological stress has been reported as a possible exacerbating factor, although its role in disease onset remains inconclusive [8]. Viral infections, including cytomegalovirus, have been suggested as potential triggers [9]. Research has linked more potential comorbidities with AA, including nutritional deficiencies (e.g., vitamin D and iron), lifestyle factors (e.g., smoking, alcohol use), systemic conditions (e.g., diabetes), autoimmune disorders (e.g., psoriasis, atopic dermatitis), and recent infections such as COVID‐19 [10, 11, 12, 13, 14, 15]. Previous studies have explored the comorbidities of AA through Mendelian randomization and meta‐analysis approaches, focusing on the relationship between AA and conditions such as atopic dermatitis (AD), depression, and iron deficiency [16, 17, 18]. However, a global trend analysis incorporating geographic and developmental factors has not yet been conducted. This study builds upon these foundational investigations by offering a public health perspective on comorbidities, providing a geographically specific analysis that can help identify high‐risk regions and inform public health interventions in these areas.

There has been no comprehensive global analysis of AA and its potential associations with comorbidities using the latest data from the Global Burden of Disease Study (GBD) 2021. Currently, global analyses of AA using the GBD data are limited to 2019 [19, 20]; even the updated analysis based on the 2021 GBD data is restricted to China [21]. Our study is the first to conduct a global analysis of AA using the GBD 2021 database. Additionally, no studies have systematically compared the relationships between AA and the Human Development Index (HDI). Furthermore, no study on GBD 2021 has performed a stratified comparison based on SDI, age, and sex to explore heterogeneity across different regions and demographic groups. We also utilized both the Estimated Annual Percentage Change (EAPC) and Average Annual Percentage Change (AAPC) to systematically present global AA trends, which have not been done in previous research. For AA, there has been no comprehensive analysis of global trends and potential associations with comorbid diseases using the latest data from the Global Burden of Disease Study (GBD) 2021 [19]. While studies on the comorbidities associated with AA have been limited to data from 2019, our research updates these findings to include data up to 2021. This study aims to address this gap by assessing global patterns of AA from 1990 to 2021 and exploring associated disease burdens. The findings may contribute to a deeper understanding of the epidemiological profile, underlying etiology, and potential comorbidities, ultimately facilitating management strategies that encompass both the physical and psychological aspects of AA.

## 2 Materials and Methods

### 2.1 Overview

Disease data for this study were obtained from the GBD 2021 database, which provides a comprehensive tool for retrieving results across multiple dimensions such as causes of death or injury, risk factors, etiology, healthy life expectancy, and population metrics. The database includes measures like deaths, years of life lost (YLLs), years lived with disability (YLDs), disability‐adjusted life years (DALYs), prevalence, and incidence. These data are systematically stratified by region, age, sex, and year (1990–2021) to ensure comparability and reproducibility. Locations are categorized based on the SDI, a composite measure reflecting development levels, which incorporates fertility rates, mean years of education, and income per capita. AA is an autoimmune disease characterized by hair loss on the scalp and other parts of the body, defined by the ICD‐10 code L63 (International Statistical Classification of Diseases, Tenth Revision, Clinical Modification). The incidence of AA was estimated using DisMod‐MR, a Bayesian meta‐regression modeling tool specifically designed to address variability and inconsistencies in epidemiological data by incorporating prior distributions, covariates, and study‐level adjustments. Prevalence estimates for AA were combined with disability weights—determined by the GBD framework based on disease severity—to calculate YLDs. The GBD methodology employs 1000 independent Monte Carlo simulations to propagate uncertainty across all estimates, with 95% uncertainty intervals (UIs) calculated using the 2.5th and 97.5th percentiles of the simulated distributions.

### 2.2 Data Collection and Estimation Framework

Data were derived from 204 countries and territories, with stratification by age and sex from 1990 to 2021. In the GBD 2021 framework, AA is classified under the skin and subcutaneous conditions. The study used a comprehensive decomposition methodology to disaggregate the incidence, prevalence, and YLDs of AA across dimensions such as age, sex, SDI, HDI, and geographic location. The 204 countries and territories included in this study were categorized by SDI into five groups—low, low‐middle, middle, high‐middle, and high—and by geographic regions: Asia, Africa, the Americas, and Europe. Trends in AA estimates were further analyzed across specific age groups: < 20, 20–24, 25–29, 30–34, 35–39, 40–44, 45–49, 50–69, and 70+ years. Given that AA is nonfatal, mortality and YLLs were not applicable, and only prevalence, incidence, and YLDs were included. YLDs were calculated by applying disability weights to the prevalence of AA at each severity level. The disability weights were derived from surveys conducted by the GBD study, ensuring alignment with global epidemiological standards. This analysis also integrated data on 22 major comorbidities associated with AA, identified through systematic literature reviews and available in the GBD 2021 dataset [13, 18, 22, 23]. The comorbidities include acne vulgaris, anxiety disorders, atopic dermatitis, bipolar disorder, chronic kidney disease, cirrhosis due to alcohol, COVID‐19, depressive disorders, diabetes mellitus, dietary iron deficiency, fungal skin diseases, inflammatory bowel disease, iodine deficiency, nonalcoholic fatty liver disease including cirrhosis, protein‐energy malnutrition, pruritus, psoriasis, rheumatoid arthritis, seborrheic dermatitis, urticaria, viral skin diseases, and vitamin A deficiency. Correlations between age‐standardized AA incidence and these comorbidities were examined to assess patterns of consistency and comorbidity across sex, SDI categories, and geographic regions. In this study, we incorporated both the SDI and the HDI to represent the development levels of each country. The HDI, a composite measure of health, education, and living standards, was sourced from the United Nations Development Programme, with scores ranging from 0 to 1 (higher scores indicate higher development levels). The SDI data were obtained from the GBD 2021 database. This study adhered to the Guidelines for Accurate and Transparent Health Estimates Reporting (GATHER) [24]. Specifically, it aligns with the 18 core principles of GATHER. The objectives, populations, and periods were clearly defined, with data inputs sourced from the GBD 2021 and inclusion criteria explicitly stated. Potential biases were assessed, and all analytical steps, including data preprocessing, model selection, and uncertainty quantification, were thoroughly described. Limitations and assumptions affecting interpretation were also addressed, ensuring compliance with the emphasis of GATHER on transparency and accuracy.

### 2.3 Statistical Analysis

Disease burden estimates were reported using age‐standardized rates, along with age‐standardized percentage changes from 1990 to 2021, accompanied by 95% UIs. To assess temporal trends in AA, the EAPC was calculated for incidence, stratified by sex, age, location, and SDI. The EAPC quantifies the annual rate of change over a specified period, estimated using the slope of a linear regression model. A positive EAPC and its lower bound > 0 indicated an increasing trend, whereas a negative EAPC and its upper bound < 0 suggested a decreasing trend; if neither condition was met, the trend was considered stable. Pearson's correlation analysis was used to examine the association between EAPCs and SDI values. All variables were presented as counts, percentages, and ratios. Joinpoint regression analysis was applied to evaluate the temporal trends of AA burden across global levels. This method identifies significant shifts in trends, segments the data accordingly, and calculates the Annual Percentage Change (APC) with 95% confidence intervals (CIs) for each subsegment. The AAPC was then computed as a summary measure for the entire study period, reflecting the overall trend direction. AAPC was calculated using the *Joinpoint* software (version 5.3.0) [25]. The Wilcoxon signed‐rank test was used to investigate differences in AA burden by sex. To compare AA burden across regions and age groups, the Kruskal–Wallis H test was applied. The Benjamini–Hochberg method was applied to adjust for multiple testing across regions, genders, and age groups. Pearson's correlation was further utilized to explore associations between AA burden and age‐standardized incidence rates (ASIRs), SDI, as well as HDI. To analyze the correlation between ASIRs of AA and its comorbidities, we used the *ggplot2* (v3.5.1), *ggrepel* (v0.9.6), and *pheatmap* (v1.0.12) R packages. All statistical analyses were performed using R (v4.4.2), with significance set at *p*‐value < 0.05.

## 3 Results

### 3.1 Global Trends

**FIGURE 1:**
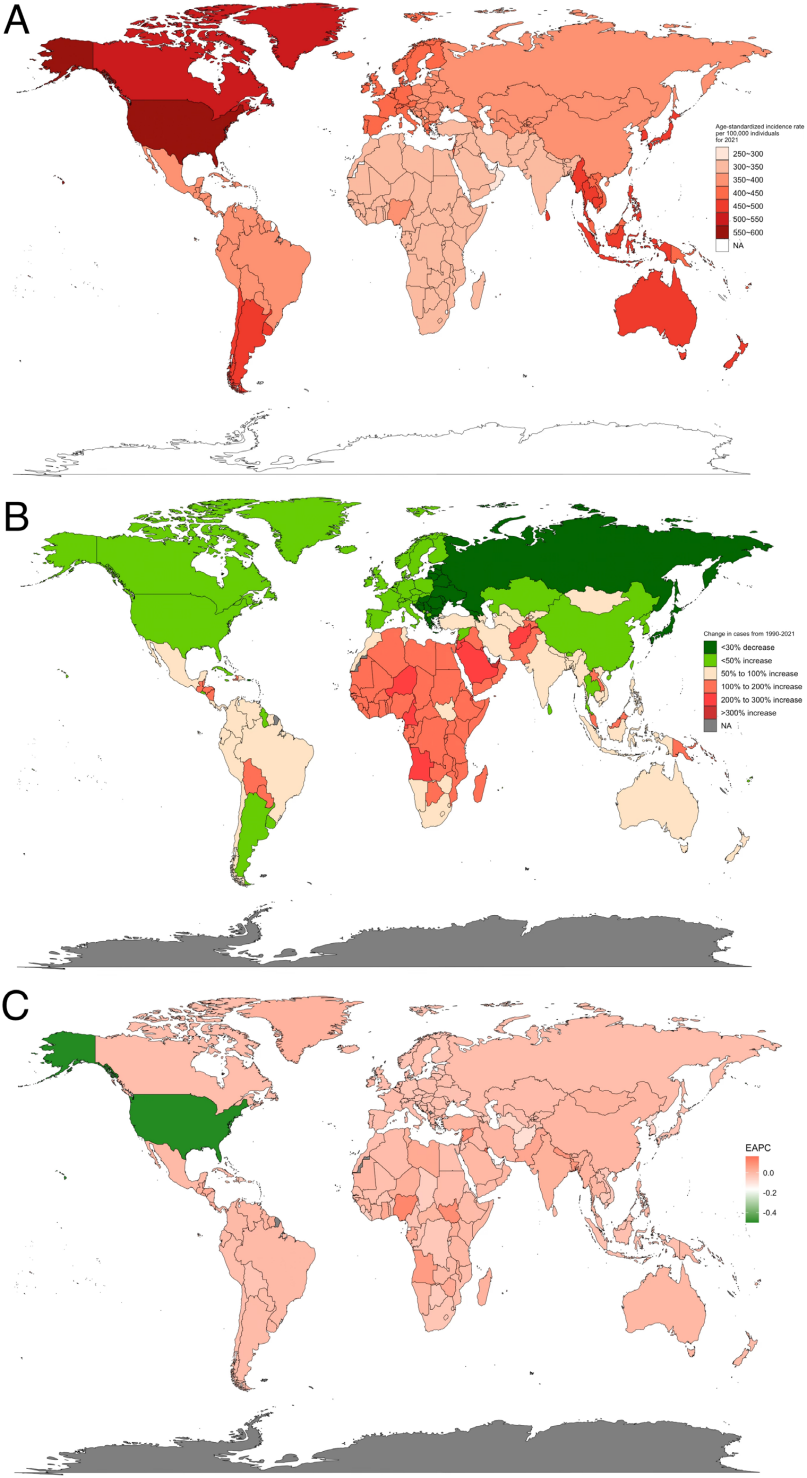
Global epidemiological characteristics of AA presented as a world map heatmap. (A) ASIR per 100,000 individuals in 2021. (B) Change in AA case numbers by country from 1990 to 2021. (C) EAPC of AA by country based on data from 1990 to 2021. AA, alopecia areata; ASIR, age‐standardized incidence rate; EAPC, estimated annual percentage change.

Globally, the incidence of AA has increased from 20.43 million (95% UI: 19.77, 21.09) in 1990 to 30.89 million (95% UI: 29.95, 31.82) in 2021. However, the ASIR decreased from 393.7 (95% UI: 381.6, 405.7) per 100 000 individuals in 1990 to 379.5 (95% UI: 368.0, 391.1) per 100 000 individuals in 2021. Between 1990 and 2021, the EAPC in incidence was −0.14 (95% CI: −0.15, −0.13), while the AAPC was −0.12 (95% CI: −0.13, −0.11). These findings indicate a slight global decline in the incidence rate of AA (Table 1). The data are presented stratified by 204 countries (Figure 1).

**TABLE 1:** Incidence of AA including number, ASIR, EAPC, and AAPC data from 1990 to 2021.

Characteristics	1990		2021		1990–2021	
	Incidence number (million; 95% UI)	ASIR (per 100 000; 95% UI)	Incidence number (million; 95% UI)	ASIR (per 100 000; 95% UI)	EAPC (95% CI)	AAPC (95% CI)
Global	20.43 (19.77, 21.09)	393.7 (381.6, 405.7)	30.89 (29.95, 31.82)	379.5 (368.0, 391.1)	−0.14 (−0.15, −0.13)	−0.12 (−0.13, −0.11)
Sociodemographic index						
High SDI	4.60 (4.46, 4.73)	486.8 (471.8, 501.1)	5.53 (5.36, 5.68)	464.9 (450.9, 479.0)	−0.23 (−0.28, −0.17)	−0.14 (−0.18, −0.09)
High‐middle SDI	4.35 (4.20, 4.49)	392.5 (379.7, 405.6)	5.54 (5.36, 5.72)	390.0 (377.2, 403.0)	−0.02 (−0.02, −0.01)	−0.02 (−0.02, −0.02)
Low SDI	1.44 (1.39, 1.49)	336.0 (325.4, 346.6)	3.40 (3.28, 3.52)	337.7 (327.1, 348.3)	0.02 (0.02, 0.02)	0.02 (0.01, 0.02)
Low‐middle SDI	3.57 (3.44, 3.69)	340.0 (329.3, 350.8)	6.59 (6.37, 6.80)	340.8 (330.0, 351.4)	0.01 (0.00, 0.01)	0.01 (0.01, 0.01)
Middle SDI	6.46 (6.23, 6.69)	384.2 (371.9, 397.3)	9.82 (9.51, 10.13)	378.5 (366.5, 390.7)	−0.05 (−0.05, −0.05)	−0.05 (−0.05, −0.05)
Region						
Africa	1.85 (1.79, 1.92)	339.8 (329.2, 350.4)	4.32 (4.17, 4.46)	341.6 (330.9, 352.5)	0.02 (0.01, 0.02)	0.02 (0.02, 0.02)
America	3.38 (3.28, 3.48)	477.8 (463.4, 491.7)	4.82 (4.68, 4.96)	441.2 (428.1, 454.5)	−0.36 (−0.44, −0.28)	−0.24 (−0.28, −0.21)
Asia	11.78 (11.37, 12.19)	381.2 (368.8, 393.8)	18.14 (17.56, 18.73)	372.0 (359.9, 384.0)	−0.08 (−0.08, −0.07)	−0.08 (−0.08, −0.08)
Europe	3.39 (3.27, 3.49)	397.6 (385.1, 410.2)	3.58 (3.47, 3.69)	394.3 (382.0, 406.8)	−0.03 (−0.03, −0.02)	−0.03 (−0.03, −0.03)
Sex						
Female	13.21 (12.79, 13.64)	514.0 (498.4, 530.0)	20.16 (19.54, 20.76)	494.8 (479.1, 510.0)	−0.15 (−0.17, −0.14)	−0.10 (−0.10, −0.10)
Male	7.22 (6.96, 7.46)	272.4 (263.5, 281.3)	10.74 (10.40, 11.08)	264.0 (255.7, 272.4)	−0.11 (−0.11, −0.1)	−0.12 (−0.13, −0.12)
Age						
< 20 years	3.88 (3.63, 4.12)	171.7 (160.8, 182.6)	4.49 (4.21, 4.77)	170.4 (159.6, 180.9)	−0.06 (−0.09, −0.02)	−0.03 (−0.04, −0.01)
20–24 years	2.43 (2.19, 2.69)	494.0 (444.2, 547.1)	2.80 (2.52, 3.10)	468.9 (421.5, 519.5)	−0.16 (−0.17, −0.15)	−0.17 (−0.18, −0.16)
25–29 years	2.91 (2.62, 3.21)	657.4 (590.9, 724.8)	3.67 (3.30, 4.05)	623.9 (560.9, 688.3)	−0.16 (−0.18, −0.15)	−0.17 (−0.18, −0.15)
30–34 years	2.68 (2.43, 2.94)	695.8 (631.2, 762.4)	4.03 (3.65, 4.42)	666.7 (604.5, 731.3)	−0.17 (−0.18, −0.15)	−0.14 (−0.16, −0.12)
35–39 years	2.23 (2.03, 2.45)	632.7 (576.2, 694.1)	3.40 (3.09, 3.74)	606.6 (551.8, 665.9)	−0.17 (−0.18, −0.16)	−0.14 (−0.15, −0.12)
40–44 years	1.57 (1.41, 1.734)	549.2 (492.3, 605.8)	2.65 (2.38, 2.93)	530.2 (475.1, 585.1)	−0.14 (−0.15, −0.13)	−0.12 (−0.13, −0.1)
45–49 years	1.10 (0.99, 1.22)	473.7 (426.4, 524.7)	2.18 (1.96, 2.42)	460.9 (414.0, 510.7)	−0.12 (−0.13, −0.10)	−0.08 (−0.1, −0.07)
50–69 years	2.95 (2.78, 3.11)	432.0 (407.7, 456.3)	6.09 (5.74, 6.43)	423.8 (399.8, 447.7)	−0.08 (−0.09, −0.06)	−0.06 (−0.07, −0.06)
70+ years	0.68 (0.64, 0.72)	335.7 (314.7, 357.7)	1.57 (1.48, 1.68)	318.5 (299.0, 339.1)	−0.33 (−0.37, −0.29)	−0.17 (−0.19, −0.15)

Abbreviations: AA, alopecia areata; AAPC, average annual percentage change; ASIR, age‐standardized incidence rate; CI, confidence interval; EAPC, estimated annual percentage change; SDI, Sociodemographic Index; UI, uncertainty interval.

In 2021, the highest ASIR values were observed in North America (United States, Canada), South America (Argentina, Chile), Southeast Asia, and Australia, where the incidence exceeded 450 per 100 000 individuals. Specifically, the United States had an ASIR exceeding 550 per 100 000 individuals, and Canada had an ASIR surpassing 500 per 100 000 individuals. Northern Europe and Western Europe also exhibited relatively high rates, with ASIRs above 400 per 100 000 individuals, followed by Russia, China, and Central America, where ASIRs were above 350 per 100 000 individuals. In contrast, Africa and the Middle East had the lowest ASIR values globally in 2021 (Figure 1A).

From 1990 to 2021, it was found that the number of AA cases in Eastern Europe and Russia has decreased, while case numbers in North America, Argentina, China, and South Korea have increased by less than 50%. Notably, a greater than 100% increase in the incidence of AA was observed in much of Africa, the Congo, and some small island nations in South Asia (Figure 1B). Concerning the EAPC in incidence globally, the United States showed the most significant decline in AA incidence (Figure 1C). Overall, countries with higher incidence rates in 2021 demonstrated a downward trend over the past 32 years, whereas countries with lower incidence rates in 2021 exhibited an upward trend. There was no significant correlation between ASIR and EAPC with latitude and longitude; these rates were more closely related to ethnic distributions.

### 3.2 Association Between AA and Socio‐Demographic Characteristics

The association between AA and various sociodemographic characteristics was assessed, including sex, age, SDI, HDI, and geographical location. The trends in incidence, prevalence, and YLDs over the past 32 years were analyzed (Figures S1–, S3). Overall, the number of cases for these characteristics has increased annually, while the ASIRs have slightly decreased. The number of cases in females is generally twice that of males. Geographically, the Americas have the highest ASIR, followed by Europe and Asia. Africa has the lowest ASIR, but it is the only region where both the EAPC and AAPC for incidence are positive (Table 1).

**FIGURE 2:**
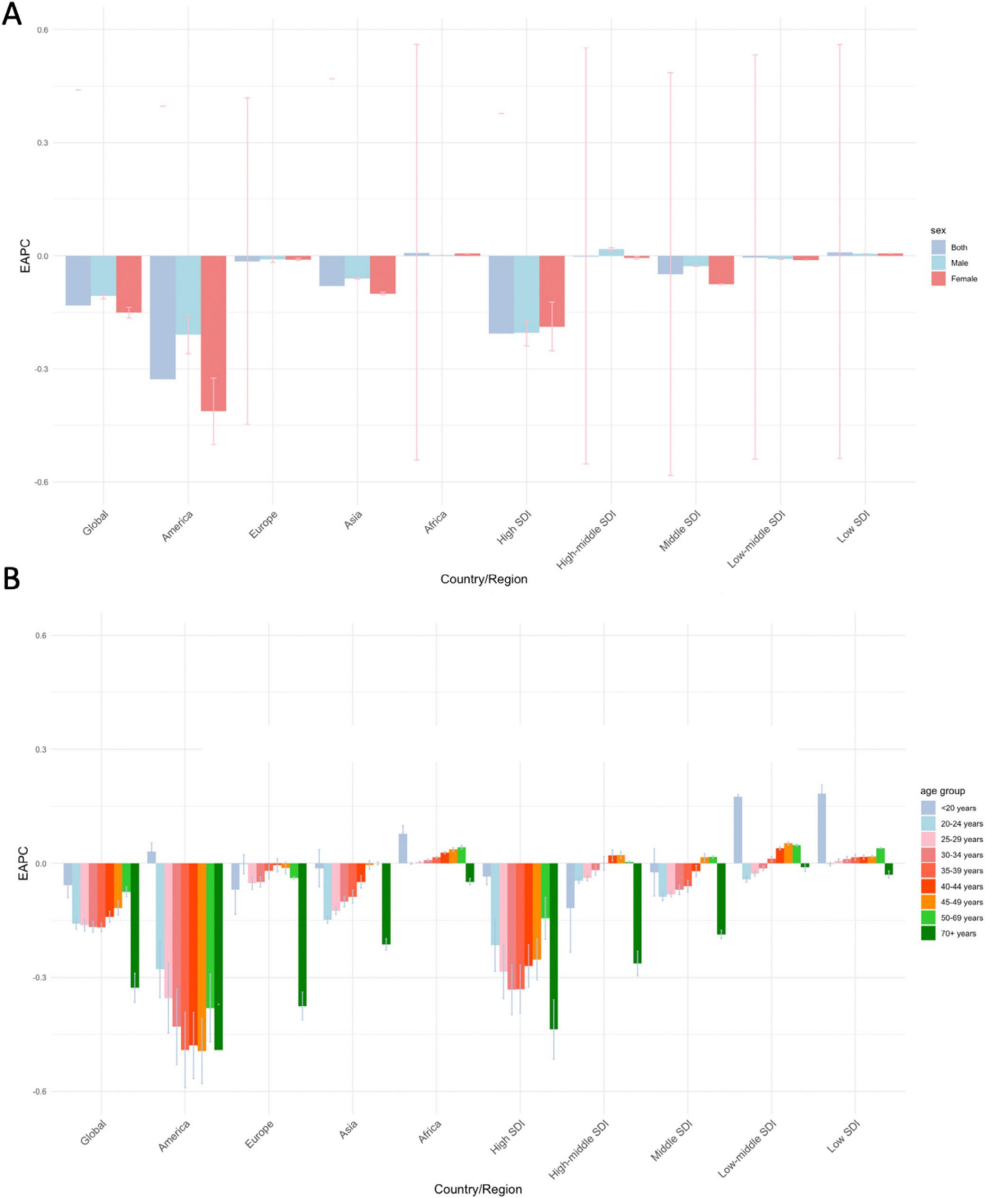
Bar plots depicting differences in EAPC of AA incidence across regions, sexes, and age groups. (A) EAPC of AA incidence by sex across various countries or regions. (B) EAPC of AA incidence by age group across multiple countries or regions. AA, alopecia areata; EAPC, estimated annual percentage change.

Regarding sex, the ASIR in females was significantly higher than in males in both 1990 and 2021, almost twice as high (*p*‐value < 0.001). However, there was no significant difference in the EAPC and AAPC for incidence between the sexes (Table 1). Age analysis revealed that individuals aged 25–40 years have the highest incidence of AA, with the highest ASIR in the 30–34 age group. The ASIR for those under 20 years or over 70 years was significantly lower (*p*‐value < 0.001), and this trend remained consistent in both 1990 and 2021. The EAPC and AAPC for incidence were negative across all age groups (Table 1). Further stratification by sex and age within each geographical region revealed the EAPC in incidence (Figure 2). Notably, the EAPC in incidence in males was higher than in females across most regions (Figure 2A). In all regions, individuals aged 70 years and above had a negative EAPC in incidence, and their EAPC values were lower than those of other age groups. In low‐SDI and low‐middle SDI regions, including Africa, individuals under 20 years exhibited a positive EAPC in incidence. Additionally, in the Americas, individuals under 20 years also showed a positive EAPC in incidence, while other regions exhibited a negative trend (Figure 2B).

**FIGURE 3:**
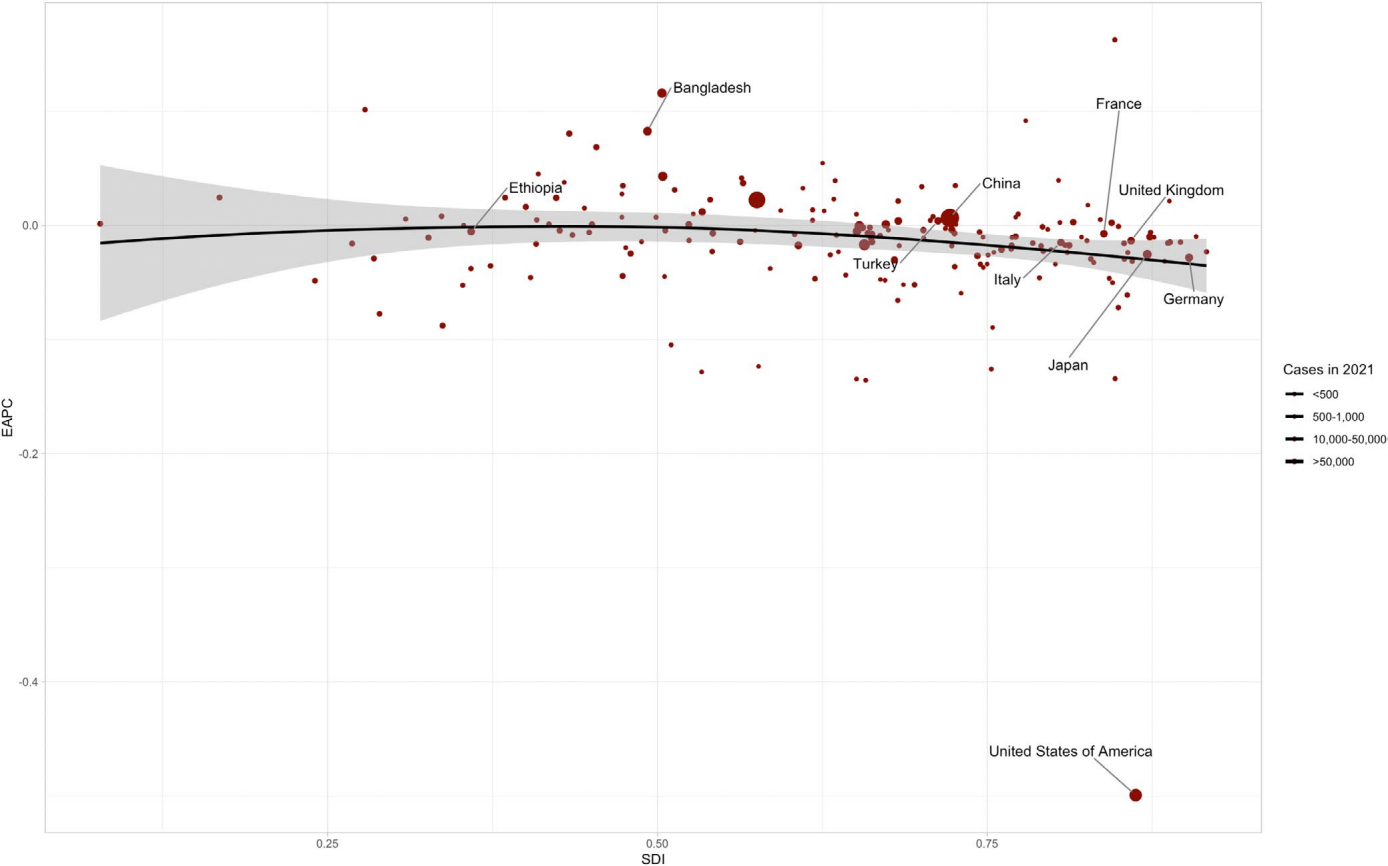
Correlation between the SDI and EAPC of AA incidence across global regions. Each point represents an SDI of a region and corresponding EAPC of AA incidence. The black LOESS regression curve, with a smoothing parameter (span) of 0.5, illustrates the nonlinear trend between SDI and EAPC. Regions are color‐coded, and labels indicate region names. AA, alopecia areata; EAPC, estimated annual percentage change; LOESS, locally estimated scatterplot smoothing; SDI, sociodemographic index.

**FIGURE 4:**
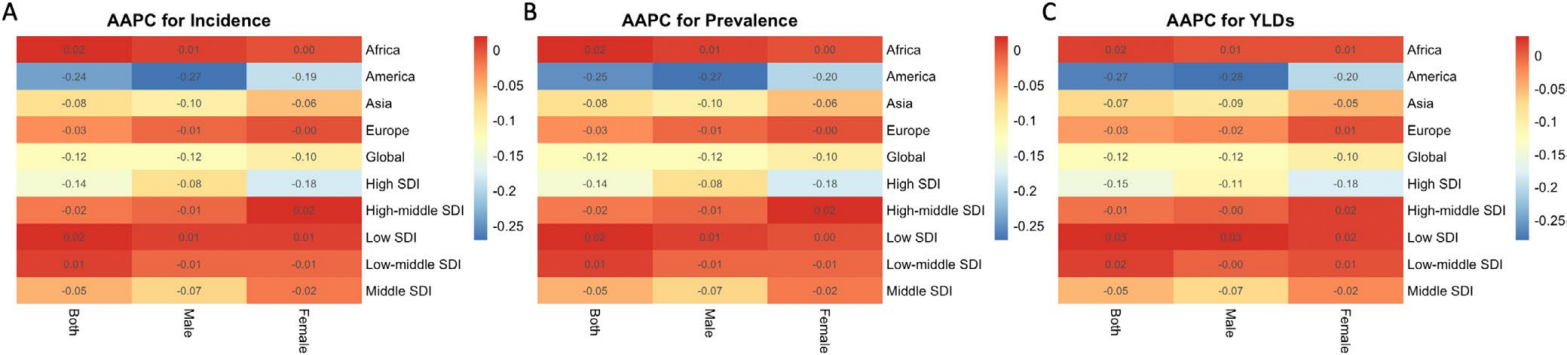
Heatmaps showing differences in the AAPC of AA incidence, prevalence, and YLDs across regions and sex groups. (A) AAPC for incidence. (B) AAPC for prevalence. (C) AAPC for YLDs. AA, alopecia areata; AAPC, average annual percentage change; YLDs, years lived with disability.

The ASIR was significantly higher in regions with higher SDI (*p*‐value = 0.017), and the EAPC in incidence was significantly lower in these regions (*p*‐value = 0.028). The AAPC was lower in regions with higher SDI but showed no significant correlation (Table 1, Figure 3). We also plotted the EAPC in incidence as a function of SDI, ASIR, and HDI (Figures 3, S4 and S5). In the SDI‐EAPC curve, we selected countries with SDI < 0.5 or SDI > 0.7 and included the ten countries with the highest incidence in each category. For countries with an SDI greater than 0.7, the United States, United Kingdom, France, Germany, Japan, Italy, China, and Turkey were selected, while for countries with an SDI less than 0.5, Bangladesh and Ethiopia were included (Figure 3). Furthermore, EAPC in incidence showed no significant correlation with HDI but was significantly negatively correlated with ASIR (*p*‐value = 0.022; Figures S4 and S5). We selected the ten countries with the highest incidence in the ASIR < 350 or ASIR > 450 categories. For countries with ASIR greater than 450, the United States, Philippines, Thailand, Indonesia, and Japan were chosen, while for countries with ASIR less than 350, Bangladesh, Pakistan, India, Egypt, and Ethiopia were selected (Figure S4). The differences in AAPC for incidence, prevalence, and YLDs across regions and sexes were analyzed. Regions with lower SDI exhibited higher AAPC for incidence, prevalence, and YLDs compared to regions with higher SDI (Figure 4).

### 3.3 Association Between AA and Comorbidities

**FIGURE 5:**
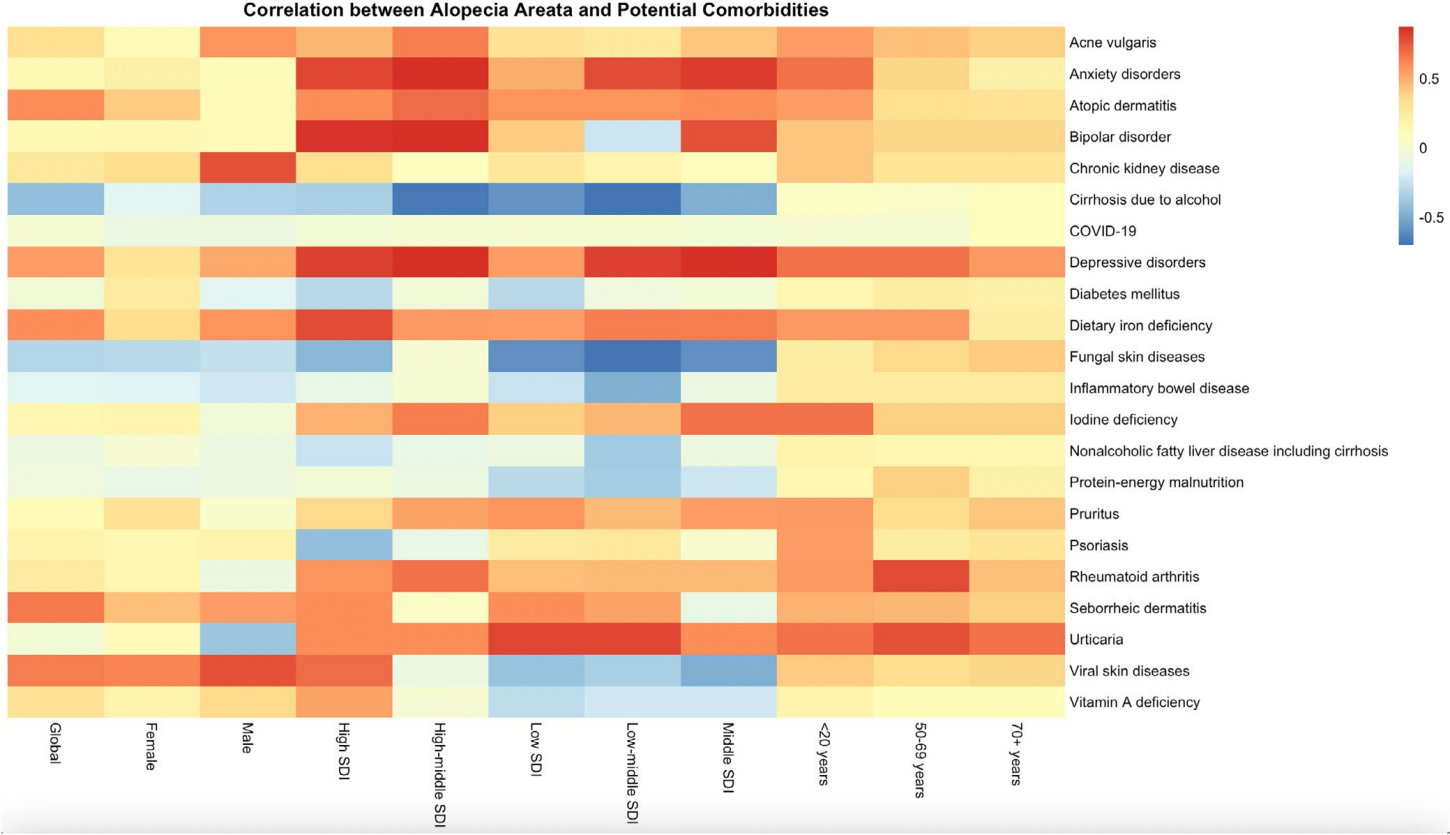
Correlation heatmap of potential comorbidities with AA incidence across regions and demographic groups (age and sex). AA, alopecia areata.

At the global level, significant associations were observed between AA and comorbidities such as AD, dietary iron deficiency, viral skin diseases, and depressive disorders, all showing strong correlations (correlation coefficient > 0.6, *p*‐value < 0.001), suggesting a robust interrelationship between these conditions and AA (Figure 5). AD demonstrated a complex correlation with AA across age groups, with a stronger association in individuals under 20 years of age, while dietary iron deficiency was more strongly correlated in those aged 50–69 years. Depressive disorders were positively correlated with AA across multiple groups, with the strongest correlation seen in high‐SDI regions, followed by females and individuals aged 50–69 years.

Regarding sex differences, acne vulgaris exhibited a stronger correlation with AA in males compared to females, while anxiety disorders were more strongly associated with AA in females than in males (Figure 5). In high‐SDI regions, anxiety disorders showed a strong correlation with AA, whereas in low‐SDI regions, some comorbidities, such as cirrhosis due to alcohol, were negatively correlated with AA. Age‐specific analysis revealed that in the under‐20 group, AD had a higher correlation with AA, while in the 50–69‐year‐old age group, conditions such as anxiety disorders and rheumatoid arthritis were more strongly associated with AA. In individuals aged 70 years and older, rheumatoid arthritis and pruritus were notably correlated with AA.

## 4 Discussion

Based on GBD 2021, this study provides a comprehensive analysis of the global trends in AA prevalence and its associations with other related diseases. It fills a significant gap in the long‐term dynamic analysis of the global AA burden and presents the most comprehensive AA prevalence map to date. Overall, we observe a slight decline in the ASIR of AA despite an increase in the absolute number of cases. This suggests that while the global burden of AA is rising, the rate of increase in incidence has slowed down.

The ASIR of AA shows a declining trend, a finding that has been corroborated by previous studies, although the underlying reasons have not been identified [20]. In general, we suppose that this decline may be closely related to improvements in medical interventions, increased public health awareness, better environmental and nutritional factors, as well as enhanced psychological support. However, further exploration through stratification by SDI, gender, and age revealed heterogeneity. In contrast to the previous study focused on localized regions [26], this global cross‐sectional comparison reveals that while the ASIR of AA in developed countries remains the highest, it is declining. Conversely, developing countries, particularly in Africa and South Asian islands, show a rapid rise in ASIR despite initially lower rates, likely due to improvements in healthcare and disease reporting. In high‐SDI regions, the EAPC in incidence is lower, suggesting a deceleration in AA growth, while lower‐SDI countries like Bangladesh and Ethiopia show higher EAPCs. The negative correlation between EAPC and ASIR (*p*‐value = 0.022) underscores the complex relationship between socioeconomic development and disease reporting. In higher ASIR countries (e.g., the US, Japan, and the Philippines), slower incidence growth may reflect better disease management and public awareness. In contrast, countries with lower ASIRs (e.g., Bangladesh, Pakistan) show increasing EAPCs, possibly due to improved disease recognition or lifestyle changes. The faster incidence rise in low‐SDI regions highlights gaps in healthcare access, education, and diagnosis, particularly in Africa and South Asian islands, suggesting emerging public health challenges and underrecognized risk factors.

Another innovation of this study is the analysis of gender and age group differences. Although gender disparities in AA incidence have been reported [1], this study validates, through global data, that the incidence in females is consistently higher than in males. Female incidence is nearly twice that of males, and this gender difference has remained consistent globally at different time points (1990 and 2021). This observation is consistent with previous studies, supporting the hypothesis that AA may be linked to sex‐specific immunological mechanisms [27]. Hormonal fluctuations and increased susceptibility to autoimmune conditions, particularly in females, have been shown to have a considerable impact [28]. Previous research has suggested that immune responses unique to females, especially those related to T‐cell activation, could contribute to the higher incidence of AA in women [29]. Nonetheless, while there is a marked gender disparity in the ASIR, no significant differences were observed in the EAPC and AAPC between males and females, indicating that the overall incidence trends of AA remain stable across genders throughout the study period.

The age‐related trends in AA incidence also provide valuable insights. Our analysis shows that individuals aged 25–40 have the highest probability of developing AA, with the highest ASIR observed in the 30–34 age group. This is consistent with the known pattern of AA onset in younger individuals [30]. In contrast, ASIR is significantly lower in those under 20 and over 70 years old (*p*‐value < 0.001), which may be due to the nature of the disease, as AA is less common in young children and the elderly. Age also interacts with geographic regions in different ways. For example, individuals aged 70 and above exhibit a negative EAPC in all regions, with lower EAPC values compared to other age groups. This may reflect factors such as lower AA incidence in the elderly population, as well as underreporting due to milder disease or late onset in this age group. In low‐SDI regions, particularly in parts of Africa, individuals under 20 show a positive EAPC, suggesting an increased awareness of the disease among younger populations.

Notably, AA may represent a distinct disease entity, a manifestation of other conditions such as AD, or a comorbid condition associated with diseases like psoriasis [31]. The diverse manifestations of AA may be driven by distinct underlying pathophysiological mechanisms. However, current research on the classification and pathogenesis of AA remains limited [32, 33, 34]. Investigating the comorbidity patterns of AA could provide valuable insights into its mechanistic heterogeneity and facilitate further studies in this area. Our study reveals strong positive correlations between AA and conditions such as AD, iron‐deficiency anemia, viral skin diseases, and depression, consistent with findings from previous studies [16, 17, 18]. Current studies suggest that the relationship between AA and atopic dermatitis is a result of both shared immune pathways and lifestyle factors. AA is traditionally considered a type 1 inflammatory disease, while AD is associated with type 2 immunity. However, studies suggest that non‐atopic AA predominantly involves type 1 immune responses, whereas AA linked to extrinsic AD shows type 2 immune activation [32]. Elevated levels of Th2 cytokines (e.g., IL‐4, IL‐5, IL‐6) and eosinophils have been noted in AA [33], and transcriptomic analysis of AA scalp lesions reveals activation of cytokine pathways such as TH1, TH2, IL‐23, and IL‐9/TH9 [34]. Both AA and AD may be influenced by factors such as smoking, alcohol consumption, psychological stress, and obesity [35]. Notably, the correlation between depression and AA is particularly pronounced in high‐SDI regions, aligning with previous reports of emotional disorders in AA patients [36]. A study from Taiwan identified a potential bidirectional relationship between AA and major depressive disorder (MDD), with both probands and unaffected siblings showing significantly increased risks, suggesting shared familial mechanisms [37]. Another study found that patients with MDD had a 90% increased risk of developing AA, while individuals with AA had a 34% increased risk of developing MDD [38]. This suggests that AA may not only be a dermatological condition but also a complex disease involving immune and emotional disturbances. In high‐SDI regions, anxiety and depression tend to show stronger correlations with AA, which may be attributed to social pressures, lifestyle factors, and healthcare intervention mechanisms in high‐income countries. Acne vulgaris, particularly in male populations, shows a stronger correlation with AA compared to females. This finding indicates that males may associate more with AA under certain conditions, warranting further investigation into gender differences and underlying mechanisms. Females show a higher correlation with anxiety disorders, while males show a lower correlation, suggesting that gender may play a role in the impact of anxiety disorders on AA, with females potentially being more susceptible.

Age‐related differences also stand out. The disease progression and comorbidity spectrum of AA vary across children, adults, and the elderly [39, 40, 41]. In this study, in individuals under 20, AD shows a high correlation, suggesting that skin diseases like AD may be more strongly associated with AA in younger populations. Among those aged 50–69, anxiety disorders and rheumatoid arthritis show stronger correlations, indicating a closer association between mental health issues, chronic diseases, and AA in middle‐aged and older adults. In the 70+ age group, rheumatoid arthritis and pruritus show stronger associations, highlighting that AA in the elderly may be linked to chronic inflammatory diseases and dermatological symptoms such as pruritus. This warrants further exploration into the impact of disease on this age group. The comorbidity spectrum of AA shows significant variation across different age groups, similar to the patterns observed in psoriasis [31]. U.S. studies reveal a stronger connection between AA and metabolic and cardiovascular diseases in adults [39], while a stronger connection exists between AA and autoimmune diseases in children, including atopic dermatitis, anemia, and obesity [40]. AA is a less common subtype of hair loss in the elderly, characterized by a high prevalence of hyperglycemia and a low prevalence of vitamin D deficiency [41]. The high burden of systemic diseases, hormonal imbalances, and nutritional deficiencies underscores the need for a comprehensive approach to hair loss management in this population. These findings align with those from the GBD database and emphasize the need for a multidisciplinary approach to managing pediatric, adult‐onset, and elderly onset AA. Therefore, developing a comprehensive comorbidome framework and adopting a patient‐specific management approach for AA comorbidities, as is done in psoriasis, is essential [31].

Despite providing global epidemiological data on AA, this study has several limitations. We believe it is necessary to further differentiate between various forms of AA, such as patchy alopecia (AT) and universal alopecia (AU). Specifically, if a patient has at least one diagnosis of AT or AU, all subsequent AA diagnoses for that patient should be classified as either AT or AU, consistent with previously published approaches [42]. The AA burden in some low‐income and lower‐middle‐income countries may have been inadequately assessed due to data sources and quality. Low diagnostic rates and limited access to treatment may result in the underreporting of AA cases, particularly in these regions. As the study primarily relies on country‐level epidemiological data, it cannot thoroughly explore racial‐ or individual‐level confounders or subgroup differences. Factors such as lifestyle, cultural background, and individual health management practices, which could significantly influence AA incidence and progression, were not fully addressed in the available data. Future research should aim to investigate these potential factors and incorporate them into burden assessments.

## 5 Conclusions

Based on the findings of this study, the global burden of AA has demonstrated a notable increase in absolute incidence from 1990 to 2021. However, the ASIR has remained relatively stable. This suggests progress in disease recognition and reporting, alongside challenges in controlling its overall prevalence. The study highlights significant demographic and regional variations, with females and individuals aged 30–34 years showing the highest disease burden. Geographically, North America, Southeast Asia, and Australia were identified as regions with the highest ASIRs, emphasizing potential disparities in healthcare access and environmental or genetic risk factors. AA could be a single disease, a manifestation of different diseases, and comorbidity. The strong associations between AA and comorbidities, such as AD, iron deficiency, and depressive disorders, underscore the multifaceted nature of the disease and its psychological and systemic impacts. These findings call for an integrated approach to managing AA that addresses both its physical and mental health consequences. The disease progression and comorbidity profile of AA varies significantly among children, adults, and the elderly. Developing a comprehensive comorbidome framework may effectively cluster patients based on shared comorbidities, enabling efficient resource allocation and targeted therapy. Future research should focus on unraveling the underlying mechanisms linking AA to its comorbid conditions, exploring regional disparities in disease burden, and identifying modifiable risk factors.

## Author Contributions

J.Z. and J.L. conceptualized the study and developed the research design. J.Z., L.L., and H.Z. contributed to the design and refinement of experimental methodologies. J.Z., M.L., and Z.Z. were responsible for data collection and acquisition of clinical and laboratory data. J.Z. and H.Z. curated and managed the data for analysis. J.Z. performed data analysis and statistical processing. J.Z. and J.L. collaboratively interpreted the study results. J.Z. and Z.Z. conducted the literature review. J.Z. drafted the initial manuscript. J.L., H.Z., M.L., and Z.Z. critically revised the manuscript for important intellectual content. J.L. provided expert guidance and reviewed all aspects of the study for intellectual consistency and rigor. All authors participated in (i) Drafting the manuscript or revising it critically for important intellectual content; (ii) Approval of the final manuscript version to be submitted; (iii) Final approval of the version to be published; and (iv) Agreement to be accountable for all aspects of the work in ensuring that questions related to the accuracy or integrity of any part of the work are appropriately investigated and resolved.

## Ethics Statement

This study did not involve the collection of new data from human participants. All analyses were conducted using secondary data from publicly available datasets from the Global Burden of Disease Study 2021. The authors confirm that the ethical policies of the journal, as noted on the journal's author guidelines page, have been adhered to. No ethical approval was required as this is a review article with no original research data.

Approval of the research protocol by an Institutional Review Board: Institutional Review Board (IRB) of Peking Union Medical Hospital (ethics number: S‐K653).

## Consent

The authors have nothing to report.

## Conflicts of Interest

The authors declare no conflicts of interest.

## Supporting information


**Figure S1.** Trends in total number of AA cases and age‐standardized incidence rates (per 100 000 persons) from 1990 to 2021, stratified by sex. Bar plots represent case numbers, and line graphs show age‐standardized rates. AA, alopecia areata.

**Figure S2.** Trends in total AA prevalence and age‐standardized prevalence rates (per 100 000 persons) from 1990 to 2021, stratified by sex. Bar plots represent prevalence numbers, and line graphs show age‐standardized rates. AA, alopecia areata.

**Figure S3.** Trends in total YLDs due to AA and age‐standardized YLD rates (per 100 000 persons) from 1990 to 2021, stratified by sex. Bar plots represent YLDs and line graphs show age‐standardized rates. AA, alopecia areata; YLDs, years lived with disability.

**Figure S4.** Correlation between ASIR and EAPC of AA incidence across global regions. Each point indicates an ASIR of a region and corresponding EAPC of AA incidence. A LOESS regression curve (black line, span = 0.5) reflects the nonlinear trend between ASIR and EAPC. Regions are color‐coded, with labels for region names. AA, alopecia areata; ASIR, age‐standardized incidence rate; EAPC, estimated annual percentage change; LOESS, locally estimated scatterplot smoothing.

**Figure S5.** Correlation between the Human Development Index (HDI) and EAPC of AA incidence across global regions. Each point represents an HDI of a region and the corresponding EAPC of AA incidence. The LOESS regression curve (black line, span = 0.5) shows the nonlinear trend between HDI and EAPC. Regions are color‐coded and labeled accordingly. AA, alopecia areata; EAPC, estimated annual percentage change; HDI, human development index; LOESS, locally estimated scatterplot smoothing.

## Data Availability

The data that support the findings of this study are openly available in the Global Burden of Disease (GBD) Study 2021 through the Institute for Health Metrics and Evaluation (IHME) website (http://ghdx.healthdata.org/).
