# Supplementary figures and images for: Global Burden of Alopecia Areata and Associated Diseases: A Trend Analysis From 1990 to 2021
Source: J Cosmet Dermatol. 2025 Feb 27;24(3):e70076. doi: 10.1111/jocd.70076 (PMC11866473; doi:10.1111/jocd.70076)

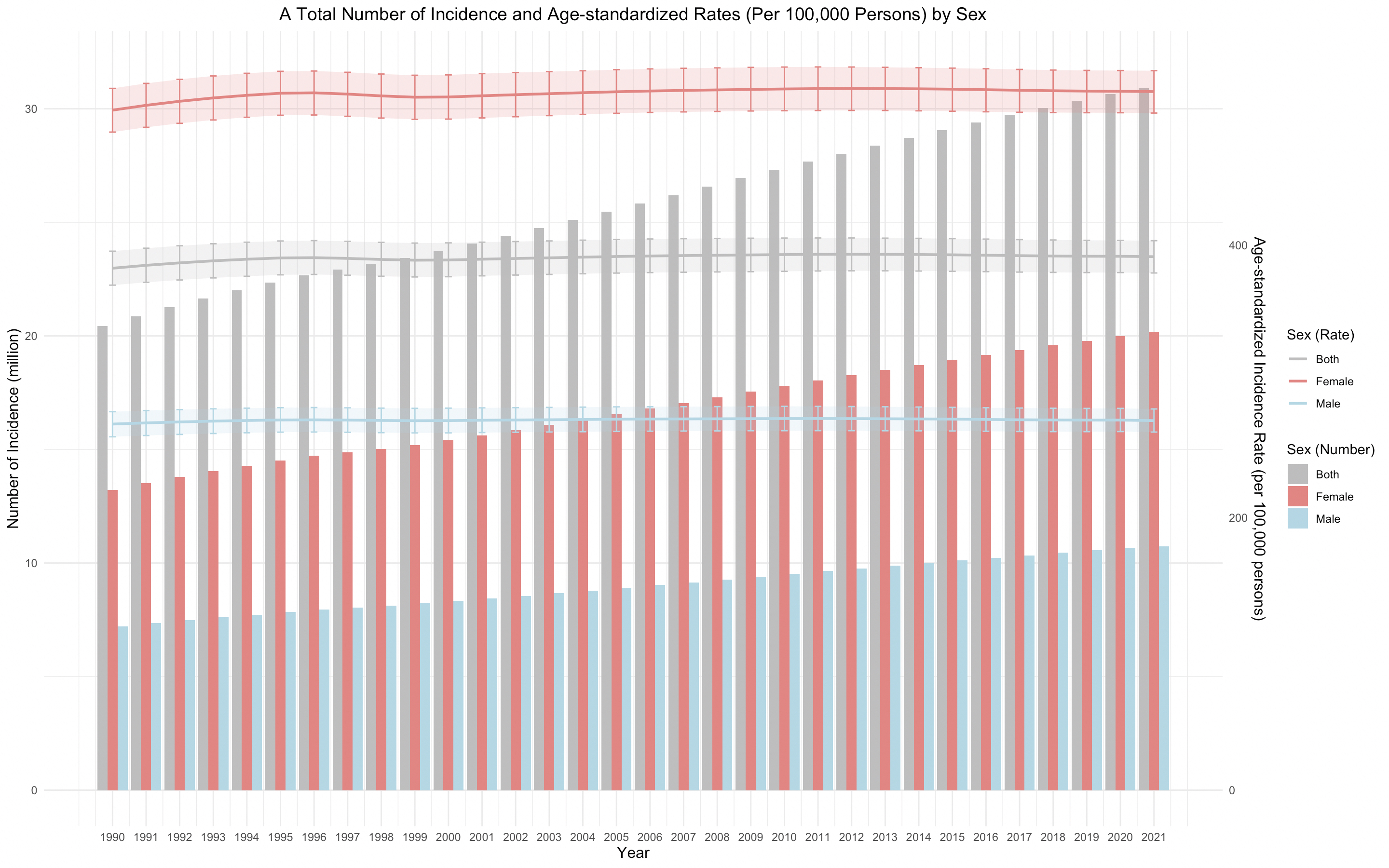

Supplement: Supplementary file 1 — Figure S1. Trends in total number of AA cases and age‐standardized incidence rates (per 100 000 persons) from 1990 to 2021, stratified by sex. Bar plots represent case numbers, and line graphs show age‐standardized rates. AA, alopecia areata. [file JOCD-24-e70076-s002.jpg]

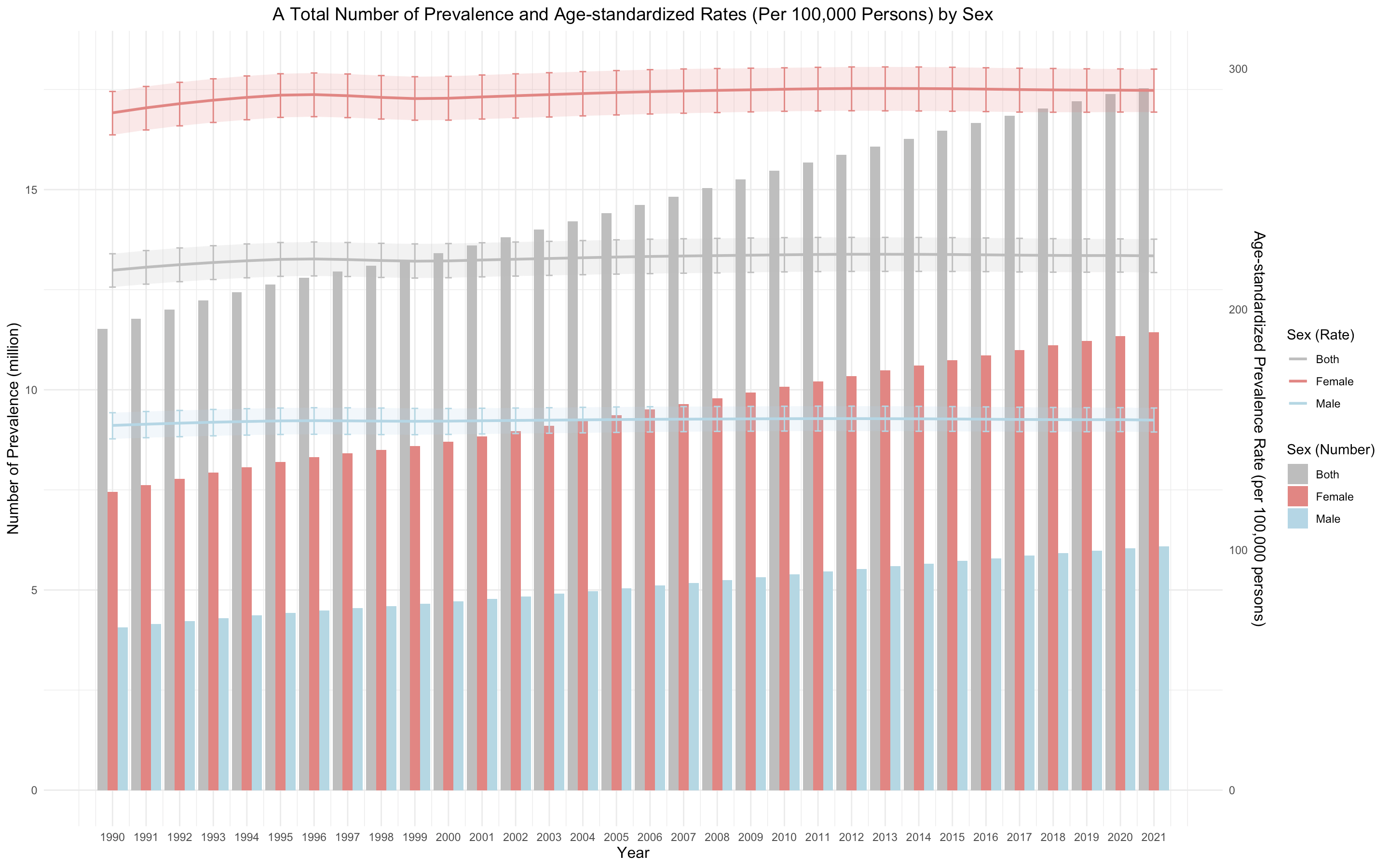

Supplement: Supplementary file 2 — Figure S2. Trends in total AA prevalence and age‐standardized prevalence rates (per 100 000 persons) from 1990 to 2021, stratified by sex. Bar plots represent prevalence numbers, and line graphs show age‐standardized rates. AA, alopecia areata. [file JOCD-24-e70076-s001.jpg]

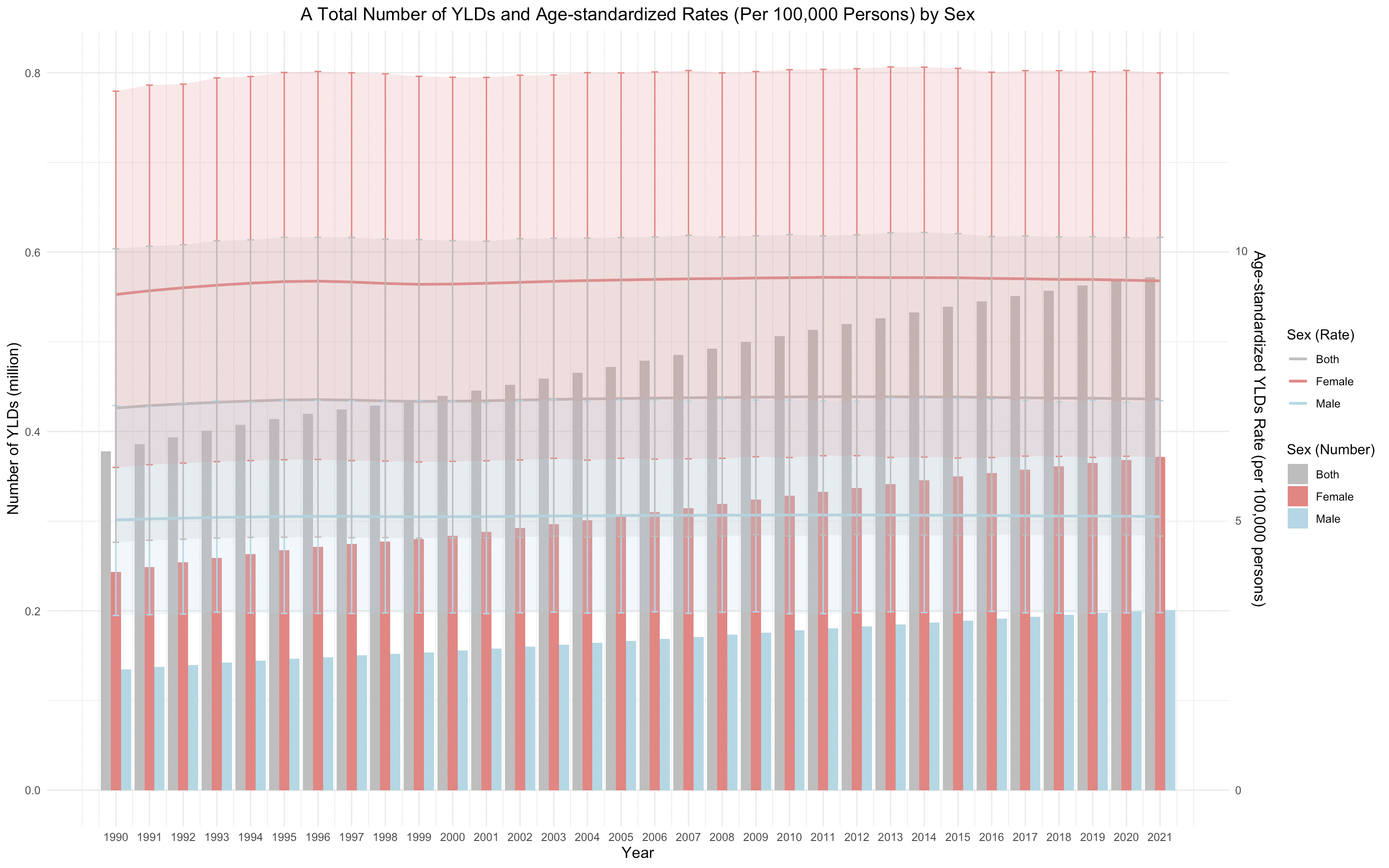

Supplement: Supplementary file 3 — Figure S3. Trends in total YLDs due to AA and age‐standardized YLD rates (per 100 000 persons) from 1990 to 2021, stratified by sex. Bar plots represent YLDs and line graphs show age‐standardized rates. AA, alopecia areata; YLDs, years lived with disability. [file JOCD-24-e70076-s005.jpg]

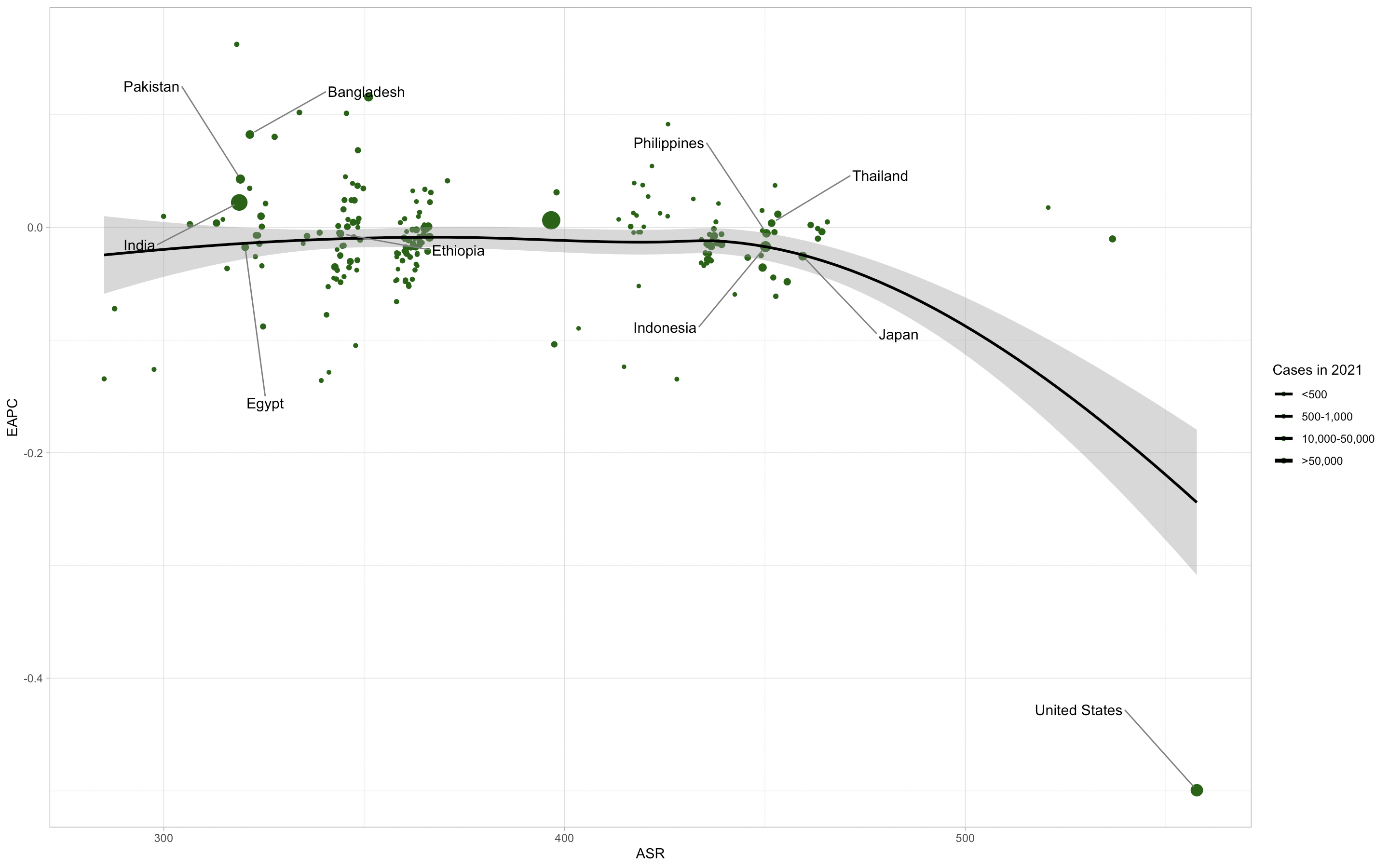

Supplement: Supplementary file 4 — Figure S4. Correlation between ASIR and EAPC of AA incidence across global regions. Each point indicates an ASIR of a region and corresponding EAPC of AA incidence. A LOESS regression curve (black line, span = 0.5) reflects the nonlinear trend between ASIR and EAPC. Regions are color‐coded, with labels for region names. AA, alopecia areata; ASIR, age‐standardized incidence rate; EAPC, estimated annual percentage change; LOESS, locally estimated scatterplot smoothing. [file JOCD-24-e70076-s004.jpg]

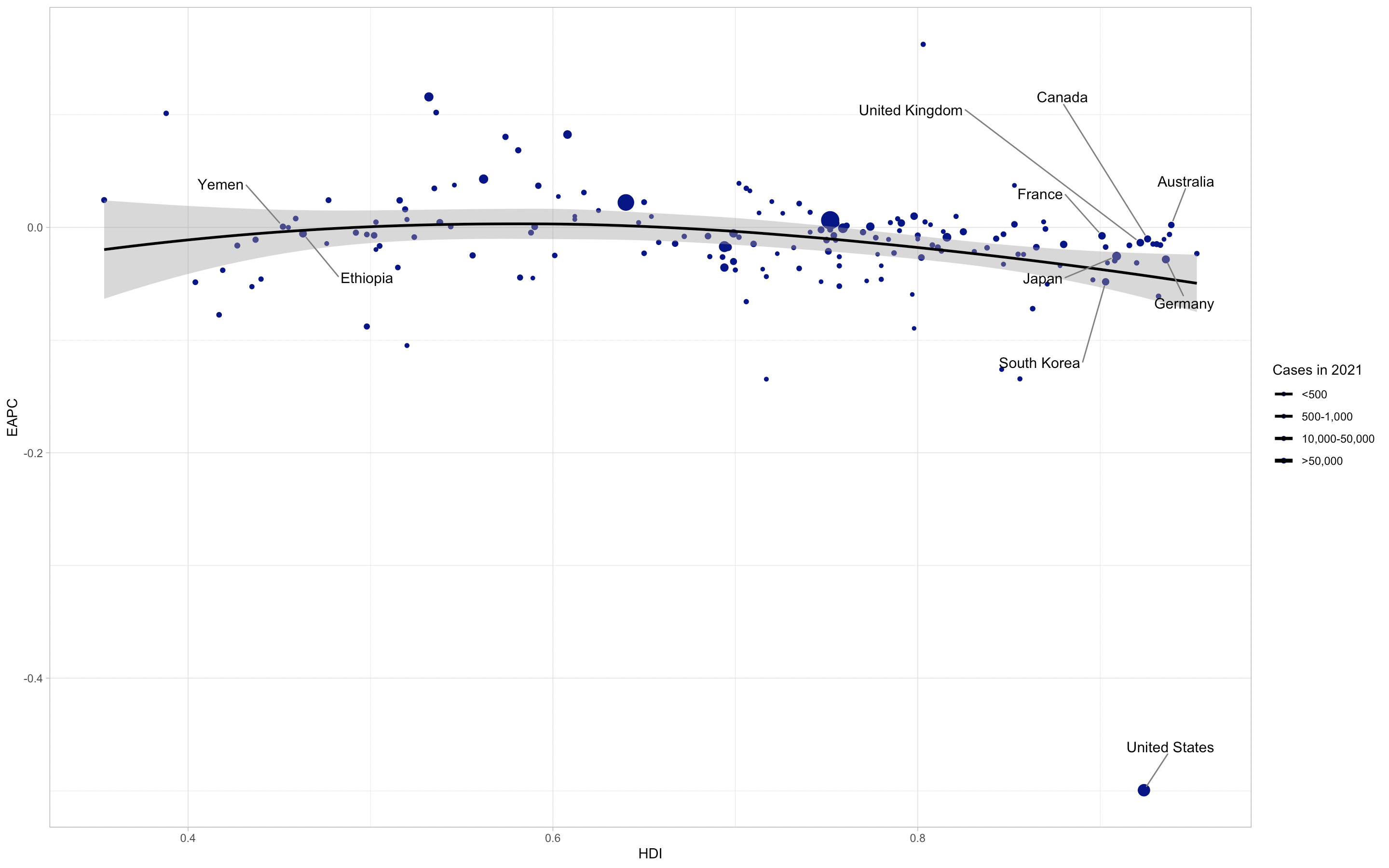

Supplement: Supplementary file 5 — Figure S5. Correlation between the Human Development Index (HDI) and EAPC of AA incidence across global regions. Each point represents an HDI of a region and the corresponding EAPC of AA incidence. The LOESS regression curve (black line, span = 0.5) shows the nonlinear trend between HDI and EAPC. Regions are color‐coded and labeled accordingly. AA, alopecia areata; EAPC, estimated annual percentage change; HDI, human development index; LOESS, locally estimated scatterplot smoothing. [file JOCD-24-e70076-s003.jpg]
